# In vivo development and single‐cell transcriptome profiling of human brain organoids

**DOI:** 10.1111/cpr.13201

**Published:** 2022-02-10

**Authors:** Shichao Huang, Fei Huang, Huiying Zhang, Yongfeng Yang, Juan Lu, Jiadong Chen, Li Shen, Gang Pei

**Affiliations:** ^1^ State Key Laboratory of Cell Biology Center for Excellence in Molecular Cell Science Shanghai Institute of Biochemistry and Cell Biology Chinese Academy of Sciences Shanghai China; ^2^ The MOE Key Laboratory of Biosystems Homeostasis & Protection and Zhejiang Provincial Key Laboratory for Cancer Molecular Cell Biology Life Sciences Institute Zhejiang University Hangzhou China; ^3^ NHC and CAMS Key Laboratory of Medical Neurobiology Center for Neuroscience and Department of Neurology of Second Affiliated Hospital MOE Frontier Science Center for Brain Research and Brain‐Machine Integration School of Brain Science and Brain Medicine Zhejiang University School of Medicine Hangzhou China; ^4^ Department of Orthopedics Surgery School of Medicine The Second Affiliated Hospital Zhejiang University Hangzhou China; ^5^ Hangzhou Global Scientific and Technological Innovation Center Zhejiang University (HIC‐ZJU) Hangzhou China; ^6^ Shanghai Key Laboratory of Signaling and Disease Research Laboratory of Receptor‐based Biomedicine The Collaborative Innovation Center for Brain Science School of Life Sciences and Technology Tongji University Shanghai China; ^7^ Institute for Stem Cell and Regeneration Chinese Academy of Sciences Beijing China

## Abstract

**Objectives:**

Human brain organoids can provide not only promising models for physiological and pathological neurogenesis but also potential therapies in neurological diseases. However, technical issues such as surgical lesions due to transplantation still limit their applications.

**Materials and methods:**

Instead of applying mature organoids, we innovatively developed human brain organoids in vivo by injecting small premature organoids into corpus striatum of adult SCID mice. Two months after injection, single‐cell transcriptome analysis was performed on 6131 GFP‐labeled human cells from transplanted mouse brains.

**Results:**

Eight subsets of cells (including neuronal cells expressing striatal markers) were identified in these in vivo developed organoids (IVD‐organoids) by unbiased clustering. Compared with in vitro cultured human cortical organoids, we found that IVD‐organoids developed more supporting cells including pericyte‐like and choroid plexus cells, which are important for maintaining organoid homeostasis. Furthermore, IVD‐organoids showed lower levels of cellular stress and apoptosis.

**Conclusions:**

Our study thus provides a novel method to generate human brain organoids, which is promising in various applications of disease models and therapies.

## Graphical abstract

Human brain organoids can provide not only promising cell models for the physiology and pathology but also potential therapies in neurological diseases. However, technical issues such as surgical lesions due to transplantation still limit their applications. In this study, we injected early human brain organoids of small size into adult mouse brain instead of transplanting mature organoids, and let premature organoids develop in vivo. By single‐cell transcriptome analysis and immunostaining, we systematically characterized these in vivo developed organoids (IVD‐organoids).

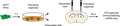

## 1 INTRODUCTION

The human brain is a highly organized structure that consists of various cell types including neurons, glia cells, and vascular cells. It is challenging to model human brain development because it requires coordinated regulation of different biochemical and physical events.^1^ Human brain organoids are stem‐cell‐derived three‐dimensional cultures that recapitulate the cellular and architectural features of a human brain, making them a promising source for transplantation therapies.^2^, ^3^ Indeed, previous studies have demonstrated that upon transplantation into adult mouse cortex, human cerebral organoids could exhibit progressive neuronal maturation and function, as well as robust host‐mediated vascularization.^4^, ^5^, ^6^, ^7^, ^8^ These findings have brightened the prospect of using brain organoid transplantation for the treatment of neurological disorders.

Previous studies have evaluated the integration and therapeutic effects of in vitro human cerebral organoids (hCOs) by transplanting them into the rodent cortex,^4^, ^5^, ^6^ but challenges still remain in the current brain organoid transplantation techniques which hinder further clinical applications. Mature cerebral organoids usually form large aggregates (up to 3–5 mm in diameter) that require larger surgical cavities for organoid transplantation, which may bring more surgical lesions to healthy tissues along the entire route of delivery, especially when transplanting organoids into deep brain regions. Thus, the surgical injury itself may lead to loss of brain function.^2^, ^9^ Therefore, there is an urgent need for new transplantation techniques that minimize injuries to the host brain.

Here instead of transplanting mature brain organoids, we injected small premature organoids into mouse brain and left them growing and differentiating to generate IVD‐organoids. Using single‐cell transcriptome analysis, we identified neuronal cells expressing striatal markers in IVD‐organoids. Compared with in vitro cultured human cerebral organoids, we also found more pericyte‐like and mature choroid plexus (ChP) cells in IVD‐organoids, which were beneficial for maintaining brain homeostasis.^10^, ^11^, ^12^, ^13^ Importantly, IVD‐organoids showed reduced levels of cellular stress and apoptosis. Our observations thus suggest promising applications of IVD‐organoids in cell therapies for neurological diseases without the risk of surgical lesions.

## 2 MATERIALS AND METHODS

### 2.1 Mice

The SCID mice were purchased from Shanghai LinChang Biotech. 4‐ to 5‐week‐old female mice were used for transplantation. All animal experiments were approved by the Institutional Animal Care and Use Committee (IACUC) of Institute of Biochemistry and Cell Biology, Shanghai Institutes for Biological Sciences, Chinese Academy of Sciences. All animals were housed in specific‐pathogen‐free animal facility and were given *ad libitum* access to food and water.

### 2.2 hPSC culture

The human induced pluripotent stem cells (hPSC) line was generated from a 66‐year‐old healthy female and characterized by iXCells Biotechnologies (catalog no. 30HU‐002). Cells were cultured on Matrigel (BD Biosciences) coated dishes in mTeSR1 medium (Stem Cell Technologies). Cultures were passaged every 5–7 days with Gentle Cell Dissociation Reagent (Stem Cell Technologies). All cells were tested negative for mycoplasma.

### 2.3 Generation of premature organoids for transplantation and mature cerebral organoids

STEMdiff™ Cerebral Organoid Kit (Stem Cell Technologies) was used for generation of premature organoids. Briefly, three different batches of hPSCs were first dissociated with Gentle Cell Dissociation Reagent (Stem Cell Technologies) and transduced by incubating with lentivirus (FUGW‐GFP) for 1 h in mTeSR1 medium containing 4 μg/ml polybrene. These GFP‐labeled hPSCs were then resuspended in EB Seeding Medium, and 9,000 cells were added into a well of 96‐well round‐bottom ultra‐low attachment plate. On day 5, EBs were transferred into a 24‐well ultra‐low attachment plate containing Induction Medium and cultured for an additional 2 days before being used for transplantation. To generate mature cerebral organoids, these premature organoids were embedded in Matrigel, transferred to Expansion Medium, and cultured for 3 days. On day 10, organoids were transferred to Maturation Medium and cultured on an orbital shaker. The medium was changed every 3 days during the maturation process.

### 2.4 Stereotaxic surgery

To perform injections, SCID mice were anesthetized with 125 mg/kg tribromoethanol (MedChemExpress) and placed in a stereotaxic apparatus. Holes were drilled on the skull to expose the dura, and three premature organoids or dissociated cells from equal number of premature organoids were injected into the striatum using a 5‐μl Hamilton syringe with a 22‐gauge needle. The coordinates were: AP: +0.5 mm, ML: +2 mm for premature organoids and −2 mm for dissociated cells. After injection, the needle was left in place for 2 min before withdrawal to avoid reflux.

### 2.5 Immunofluorescence staining

For cultured organoids, residual Matrigel was removed by washing with PBS. Organoids and mouse brains were fixed with 4% PFA in PBS overnight at 4℃ and submerged in 30% sucrose for 2–3 days until samples sank. Then, organoids or mouse brains were embedded in OCT and cryosectioned at 20 or 30 μm, respectively. Antigen retrieval was performed with Citrate Antigen Retrieval Solution (Beyotime) at 95°C for 20 min. Slides were incubated overnight at 4°C with primary antibodies diluted in PBS containing 0.3% Triton X‐100 and 3% donkey serum. After three washes, slides were incubated with secondary antibodies at room temperature for 1 h. Primary antibodies used were as follows: MAP2 (rabbit, Millipore AB5622, 1:500), GFAP (rabbit, DAKO Z033401, 1:1000), STEM121 (mouse, Takara Y40410, 1:500), Iba1 (rabbit, Wako 019‐19741, 1:500), CD31 (rabbit, Beyotime AF0099, 1:200), PDGFRβ (rat, Thermofisher 14‐1402‐81, 1:500), Zbtb20 (rabbit, Proteintech 23987‐1‐AP, 1:200), TTR (mouse, R&D MAB7505, 1:500), NESTIN (mouse, Millipore MAB5326, 1:500), DCX (goat, Santacruz sc‐8066, 1:200), LHX2 (rabbit, Abcam ab184337, 1:500), TBR1 (rabbit, Abcam ab31940, 1:500), Brychyury (rabbit, Abcam ab20680, 1:500), NGN2 (rabbit, Cell Signaling Technology 13144, 1:200), MASH1 (rabbit, Abcam ab211327, 1:100), FoxG1 (rabbit, Abcam ab18259, 1:100), CA12 (rabbit, Abcam ab195233, 1:100), and cleaved caspase 3 (rabbit, Cell Signaling Technology 9661, 1:200). Secondary antibodies used were as follows: donkey anti‐rabbit cy3 (Jackson ImmunoResearch 711‐165‐152, 1:1000), donkey anti‐mouse Alexa 647 (Jackson ImmunoResearch A31571, 1:1000), donkey anti‐mouse cy3 (Molecular Probes 715‐165‐150, 1:1000), donkey anti‐rat cy3 (Jackson ImmunoResearch 712‐165‐150, 1:1000), and donkey anti‐goat cy3 (Jackson ImmunoResearch 705‐165‐147, 1:1000). All images were scanned using Olympus FV10i confocal microscope.

### 2.6 scRNA‐seq library preparation and sequencing

Two months after injection of premature organoids, mice were anesthetized with chloral hydrate and perfused with cold PBS. Brain was immediately removed from the skull and coronally sectioned with an ice‐cold brain slicer matrix. GFP‐positive regions in brain slices were rapidly dissected out using a fluorescence stereo microscope, digested for 30 min at 37°C with enzymatic dissociation medium (1 U/ml dispase, 2.5 U/ml papain, and 250 U/ml DNase I; all from Worthington), and mechanically triturated. The cell suspension was passed through a 40 μm cell strainer, and debris was removed by 22% Percoll (Sigma‐Aldrich) density gradient centrifugation. The 3’ scRNA‐seq library construction was performed on a Chromium Controller (10× Genomics) with the Single Cell 3’ v2 kit following the manufacturer's instructions. The target capture rate was 8,000 cells per sample. Final libraries were sequenced on a HiSeq X Ten system (Illumina) as paired‐end 150‐bp reads.

### 2.7 scRNA‐seq data processing

Sequencing reads were aligned to mixed reference genome containing human (hg38), mouse (mm10), and GFP sequences using STAR in Cell Ranger 3.1.0 with default parameters. After removing poorly mapped reads and PCR duplicates, reads that were uniquely mapped to human transcriptome or GFP sequence and contained valid cell barcodes and unique molecular identifiers (UMIs) were used to generate the gene‐barcode matrix. Cells with less than 200 detected genes, or more than 20% mitochondrial transcripts, or no GFP reads were discarded. After filtering, gene expression in each cell was calculated as transcripts per 10,000 transcripts (TP10K), representing the fraction of each gene's UMI count with respect to total UMIs in the cell and multiplied by 10,000. Only genes expressed in at least three cells were retained. The resulting digital expression matrix was log‐transformed before downstream analyses with Seurat (v3.2.0). The top 2,000 highly variable genes were used to perform principal component analysis (PCA). Dimension reduction was implemented by uniform manifold approximation and projection (UMAP) using 1st to 25th principal components (PCs). Cell clusters were identified based on the Louvain clustering. Differentially expressed genes (DEGs) in each cluster were identified using the “FindAllMarkers” function with default parameters. Cell‐type annotation was performed similarly to previous reports.^14^, ^15^

### 2.8 Integration analysis with published organoid scRNA‐seq data

Integration analysis of IVD‐organoids and hCOs (GSE134049) was performed using the Seurat 3 integration pipeline. Canonical correlation (CC) analysis with the top 10 CC components was used to reduce the batch effects between two datasets. DEGs between two datasets in the corresponding clusters were identified using the “FindMarkers” function with the MAST algorithm. GO term analysis of DEGs was performed using the clusterProfiler package.^16^ PCA analysis was performed on ChP cells in IVD‐organoids, together with mature ChP cells, immature ChP cells, neuronal cells, and stroma cells in ChP organoids (GSE150903). To estimate the levels of apoptosis (HALLMARK: M5902), glycolysis (GO: 0061621), hypoxia (GO: 0001666), and endoplasmic reticulum (ER) stress pathways (GO: 0034976), average expression levels of genes in these pathways were calculated. Two‐sided Wilcoxon's rank‐sum test was used to determine the statistical significance.

### 2.9 Statistical analysis

Statistical analyses were performed using GraphPad Prism 6 and R (3.6.1). Two‐tailed unpaired Student's *t*‐test was used unless stated otherwise. Data were presented as a mean ± s.e.m. (standard error of mean). *p*‐values in multiple tests were adjusted by false discovery rate (FDR) Benjamini–Hochberg method, and FDR <0.05 was defined as statistical significance.

## 3 RESULTS

### 3.1 In vivo development of human brain organoids in mouse brain

**FIGURE 1:**
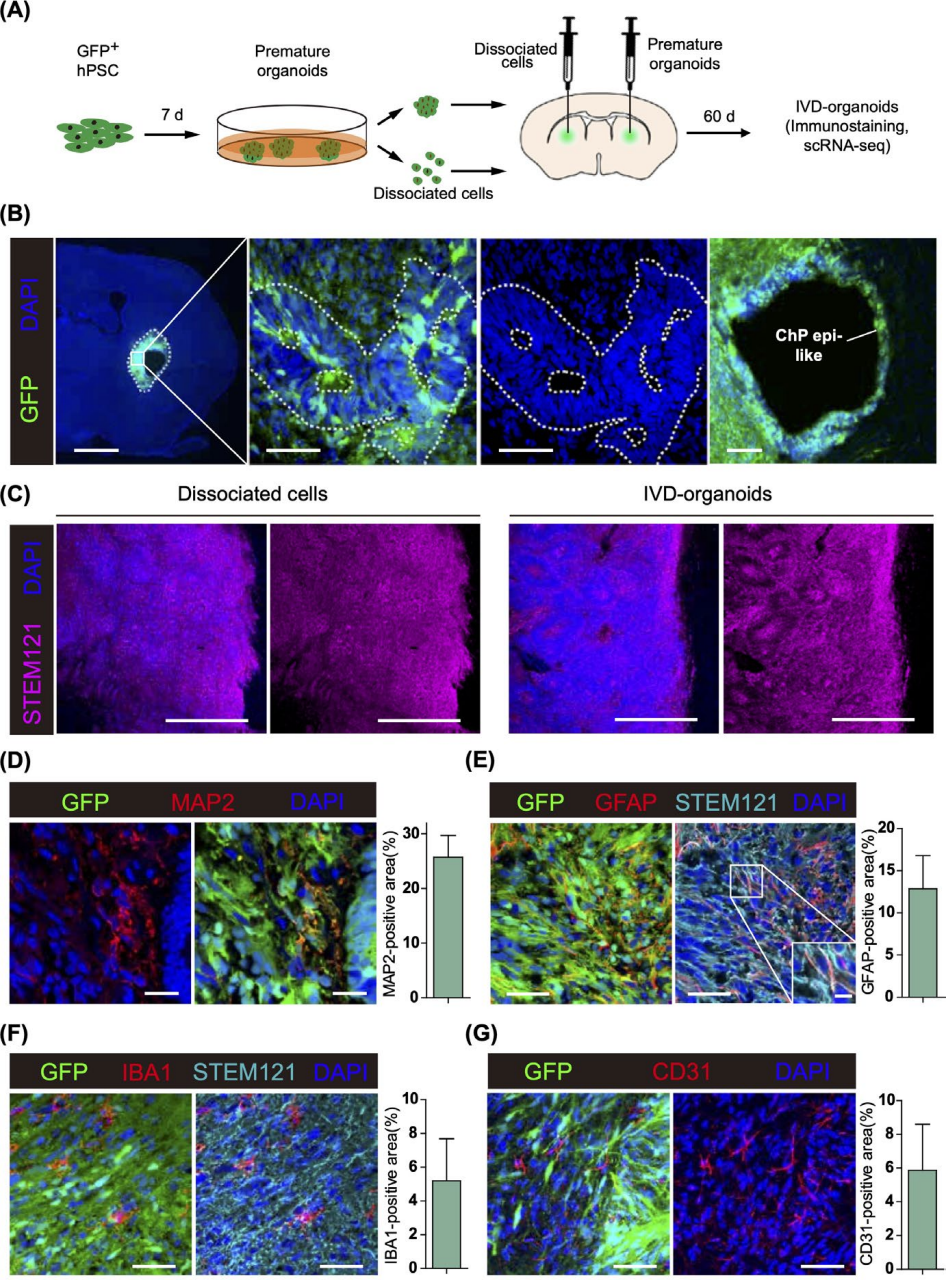
In vivo development of human brain organoids in mouse brain. (A) Experiment procedure for premature organoid generation and injection into mouse corpus striatum. (B) IVD‐organoids were immunostained for GFP and DAPI. Left, coronal section showing IVD‐organoids survived in mouse corpus striatum. Scale bar, 1000 μm. Middle, enlarged image showing the ventricle zone‐like structure. Scale bars, 50 μm. Right, enlarged image showing the choroid plexus epithelium‐like structure. Scale bar, 100 μm. (C) Comparation of grafted cells (left) and IVD‐organoids (right). Scale bars, 500 μm. (D) Immunostaining of MAP2 in IVD‐organoids. Scale bars, 20 μm. (E) Immunostaining of GFAP and STEM121 in IVD‐organoids. Scale bars, 50 μm and 10 μm (high‐magnification images). (F) Immunostaining of IBA1 in IVD‐organoids. Scale bars, 50 μm. (G) Immunostaining of vascular marker CD31 in IVD‐organoids. Scale bars, 50 μm

To trace the premature organoids after transplantation, we first performed lentiviral infection to obtain GFP‐expressing hPSCs, which were then differentiated into premature organoids. After 7 days of in vitro culture, these premature organoids were transplanted into corpus striatum of immune‐deficient mice by stereo injection (*n* = 3). As a control, we also injected dissociated cells from equal number of premature organoids into the corpus striatum of the opposite side of the same mice (Figure 1A). Day‐7 premature organoids were also analyzed by immunostaining for dorsal telencephalic marker NGN2, dorsal cortical marker LHX2, ventral telencephalic markers MASH1 and NKX2.1, preplate marker TBR1, forebrain marker FoxG1, neural stem/progenitor cell marker NESTIN, immature neuronal marker DCX, choroid plexus marker TTR, pericyte marker PDGFRβ1, and mesodermal marker Brachyury. We only observed NESTIN‐positive cells, indicating the emergence of neural stem/progenitor cells instead of more differentiated cells in these day‐7 premature organoids (Figure S1A).^17^ Two months after injection, we dissected the recipient mice and found that the GFP‐labeled IVD‐organoids exhibited robust survival and ventricle zone (VZ)‐like structures, near which large cavities surrounded by GFP‐positive ChP epithelium‐like cells could often be found (Figure 1B). Such structures were not observed in the corpus striatum of the opposite side where dissociated cells were injected, suggesting that the organoid‐like structures were specific to IVD‐organoids (Figure 1C and Figure S1B). Nevertheless, neuronal and astrocytic differentiation could be robustly observed in both IVD‐organoids and dissociated cells as indicated by neuron marker MAP2 and astrocyte marker GFAP (Figure 1D, E and Figure S1C, D). Interestingly, although IBA1‐expressing microglia cells were also found in IVD‐organoids and dissociated cells, they did not express GFP or human‐specific marker STEM121, suggesting these microglia cells were migrated from host tissues (Figure 1F and Figure S1E). GFP‐negative CD31‐expressing microvasculature‐like structures of host origin were also observed in the injection sites, indicating the formation of vascular network which may contribute to the survival and long‐term development of IVD‐organoids and dissociated cells (Figure 1G and Figure S1F).

### 3.2 Single‐cell transcriptome analyses of IVD‐organoids

**FIGURE 2:**
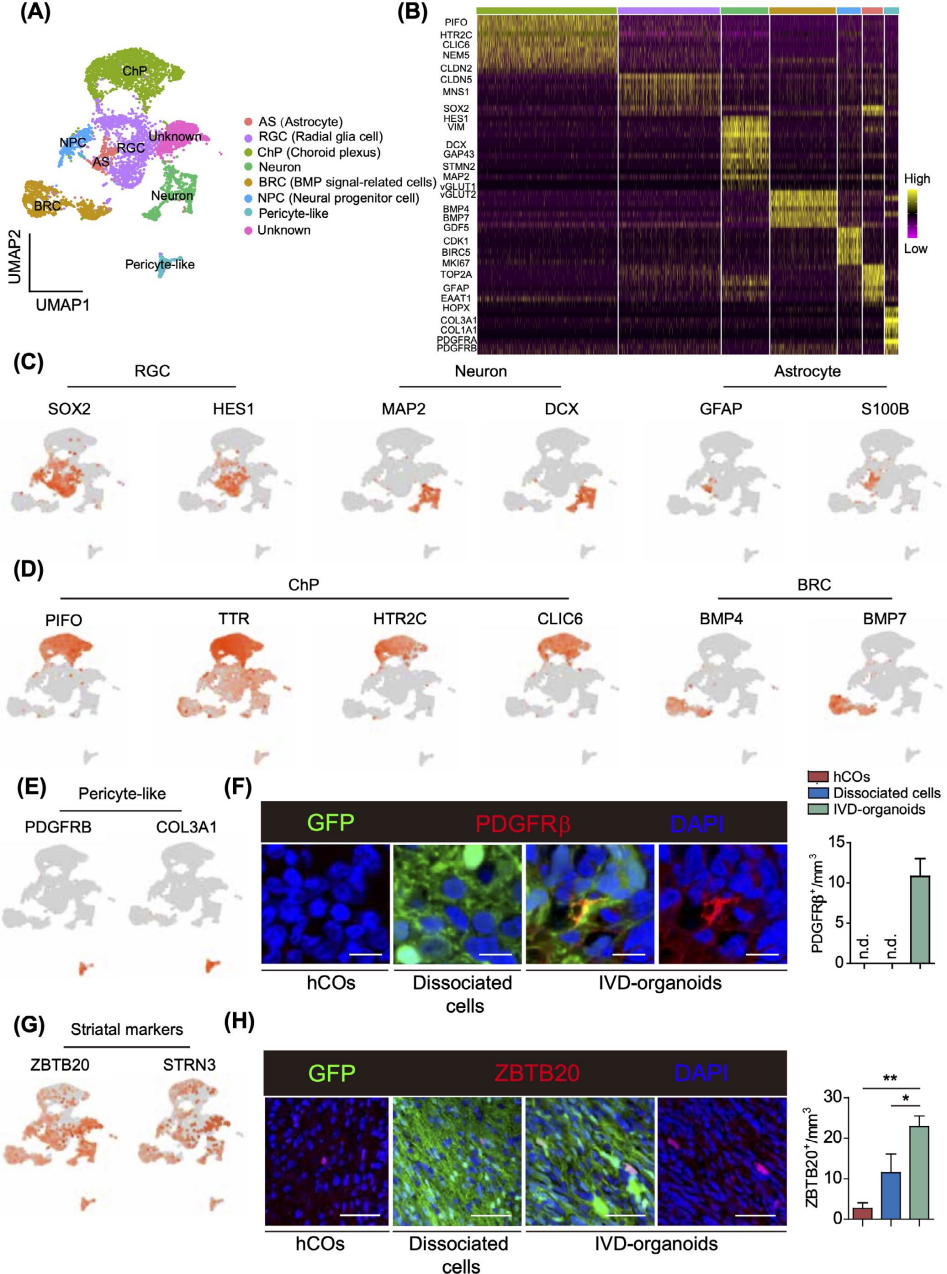
Single‐cell transcriptome analysis of IVD‐organoids. (A) UMAP dimension reduction plots of IVD‐organoids. Colored by cell‐type annotation. BRC, BMP signal‐related cell; ChP, choroid plexus; RGC, radial glial cell; AS, astrocyte; NPC, neural progenitor cell. (B) Heatmap showing relative expression levels of cell‐type markers. (C) Expression patterns of marker genes in neuron‐related clusters. (D) Expression patterns of marker genes in supporting cells. (E) Expression patterns of marker genes in pericyte‐like cells. (F) Immunostaining of PDGFRβ in IVD‐organoids, dissociated cells and D60 hCOs. Scale bars, 10 μm. Quantification results are presented as mean ± s.e.m. (*n* = 3 for transplanted mice and cultured hCOs). (G) Expression patterns of striatal markers. (H) Immunostaining of ZBTB20 in IVD‐organoids, dissociated cells and D60 hCOs. Scale bars, 50 μm. Quantification results are presented as mean ± s.e.m. (*n* = 3 for transplanted mice and cultured D60 hCOs). ∗∗*p* < 0.01, ∗*p* < 0.05, unpaired two‐tailed *t*‐test

To gain further insight into the cell‐type composition in IVD‐organoids, we dissected out the GFP‐positive IVD‐organoids under a fluorescence stereo microscope two months after transplantation and performed single‐cell transcriptome analysis. In total, we captured 6,131 GFP‐expressing human cells, among which 8 subsets of cells were identified by unbiased clustering (Figure 2A, B). The majority of identified cell types in our IVD‐organoids were also reported in stage‐matched in vitro hCOs,^18^ including radial glial cells (RGCs), neurons, and astrocytes (Figure 2C). We also detected in IVD‐organoids supporting cells that were beneficial for neurogenesis and cell survival,^19^ such as ChP cells and BMP signal‐related cells (BRCs, GO:0030509, possibly be related to certain neural precursor cells during brain development) (Figure 2D). Unexpectedly, we found a pericyte‐like cluster expressing PDGFRβ and COL3A1 (Figure 2E). Immunostaining further verified the existence of PDGFRβ‐positive cells in IVD‐organoids, which were not detected in stage‐matched in vitro hCOs and transplanted dissociated cells (Figure 2F). These results suggested that perivascular lineage differentiation, which is important for the neurovascular formation during brain development,^20^ could be induced in the grafted premature organoid. IVD‐organoids thus exhibited increased cell‐type diversity than hCOs, with more supporting cells generated.

Defined culture conditions guide the in vitro development of organoids representative for certain brain regions.^3^, ^21^ In order to clarify the regional identity of IVD‐organoids, we analyzed the expression profile of brain regional signature genes in IVD‐organoids. In contrast to hCOs which mainly recapitulated the development of cortex,^2^, ^18^ we found that cortical markers were hardly expressed in IVD‐organoids (Figure S2A). Instead, we unexpectedly found that many cells in IVD‐organoids expressed striatal markers ZBTB20 and STRN3. Consistently, we observed more ZBTB20‐positive cells in IVD‐organoids than in hCOs and dissociated cells by immunostaining (Figure 2G, H). These ZBTB20‐positive cells were distributed sparsely in the IVD‐organoids instead of in the lateral ganglionic eminence (LGE)‐like pattern, probably due to the relative short period of time between transplantation and detection^22^, ^23^, ^24^ (Figure S2B). These results thus indicated that IVD‐organoids exhibited a striatal identity, likely guided by their brain microenvironment.

### 3.3 Efficient generation of ChP cells in IVD‐organoids

**FIGURE 3:**
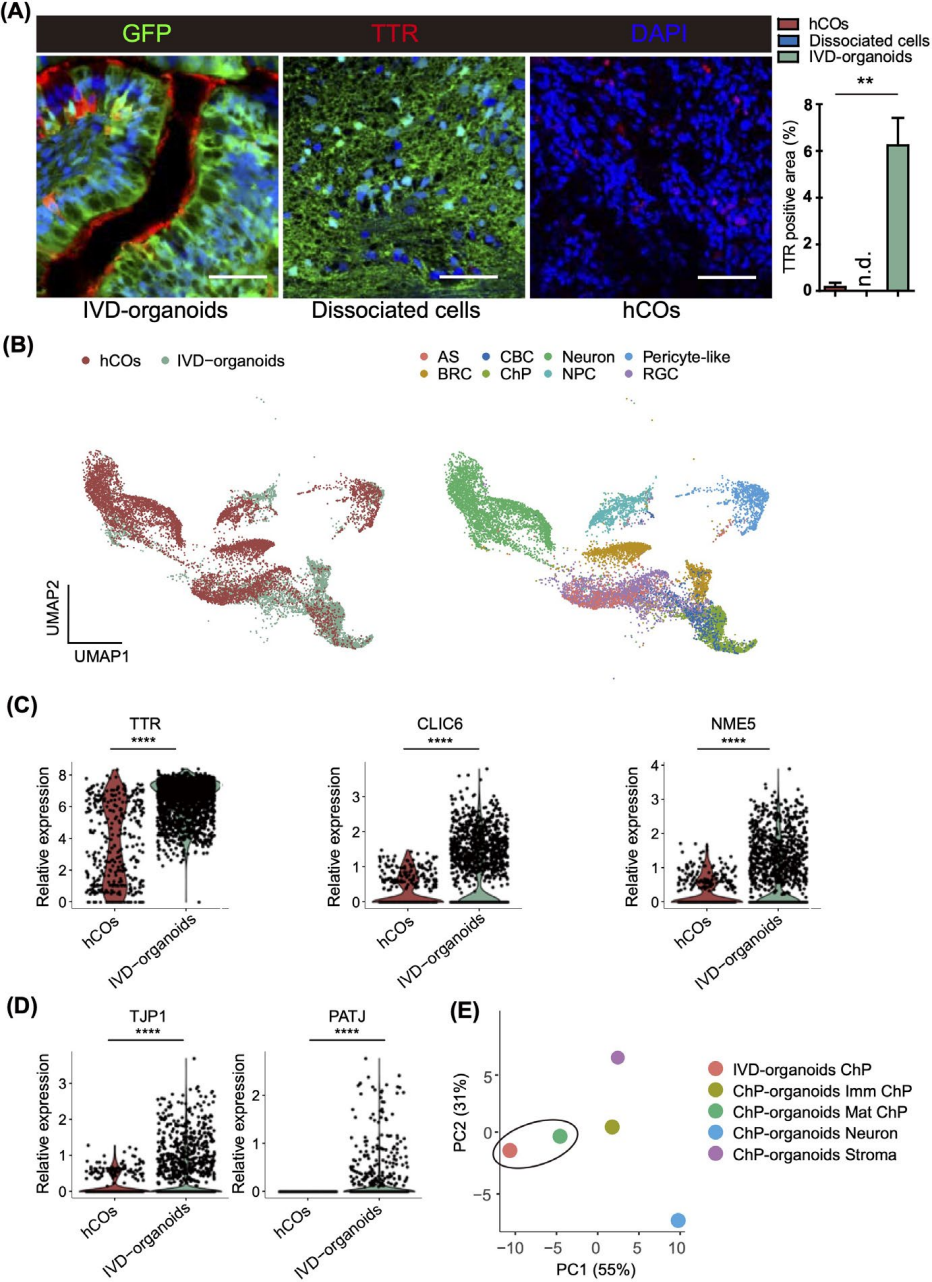
ChP cells are efficiently generated in IVD‐organoids. (A) Immunostaining of TTR in IVD‐organoids, dissociated cells and day 60 hCOs. Scale bars, 50 μm. Quantification results are presented as mean ± s.e.m (*n* = 3 for transplanted mice, dissociated cells and cultured hCOs). (B) Integrated UMAP plots showing joint clustering of IVD‐organoids and hCOs. Colored by samples (left) and cell types (right). (C) Violin plots showing expression levels of mature ChP markers in hCOs and IVD‐organoids. (D) Violin plots showing expression levels of tight junction markers in hCOs and IVD‐organoids. (E) PCA analysis of the ChP cell cluster in IVD‐organoids and various cell clusters in ChP organoids

In the IVD‐organoids, ChP cells were one of the major cell subsets identified by our single‐cell transcriptome analysis (Figure 2A). ChP is important for cerebrospinal fluid production, neuronal survival, and brain homeostasis,^11^ and ChP cilia play important roles in the regulation of brain development and neuroinflammation.^25^ Indeed, we observed expression of cilia markers such as FOXJ1, RSPH1, and ROPN1L in the ChP cluster (Figure S2C). Consistent with the single‐cell transcriptome analysis, we observed by immunostaining ChP epithelium‐like structures with transthyretin (TTR)‐positive areas around ventricles of IVD‐organoids, but not in hCOs and dissociated cells (Figure 3A and Figure S2D). Examination of regional markers revealed that most ChP cells expressed telencephalic markers instead of hindbrain markers, suggesting a telencephalic origin of the ChP cells in IVD‐organoids (Figure S3A).

To further investigate the identity of ChP cells, we performed integrated analysis of single‐cell transcriptome data of IVD‐organoids and previously reported stage‐matched in vitro hCOs.^15^ We found that ChP cells from IVD‐organoids were co‐clustered with cilium bearing cells (CBCs) in hCOs (Figure 3B). ChP cells in IVD‐organoids expressed higher levels of mature ChP markers (TTR, CLIC6, and NME5) compared with CBCs in hCOs (Figure 3C), suggesting the generation of mature ChP cells in IVD‐organoids. Indeed, we observed in IVD‐organoids but not in hCOs robust expression of marker genes for tight junctions (TJP1, PATJ, CLDN1, and CLDN2) (Figure 3D and Figure S3C), a feature of mature ChP cells which is important for ChP to form selective brain barriers to protect central nervous system from harmful substances. Consistent with their functions in secreting cerebrospinal fluid (CSF), ChP cells in IVD‐organoids also highly expressed CSF secretion associated transporters, including APOD (CSF lipoprotein), PARK7 (parkinsonism associated deglycase), and CA2/CA12 (CSF secretion enzymes) (Figure S3B–D). These observations were in agreement with the crucial roles of ChP cells in controlling nutrient transport and CSF production. We further performed integrated analysis of single‐cell transcriptome data of IVD‐organoids and previously reported human ChP organoids^26^ and found that ChP cells in IVD‐organoids were most similar to the mature ChP cells in ChP organoids (Figure 3E). Although these differences might be partly owing to the variability among different organoid cultures, our findings at least indicated efficient generation of mature ChP cells in IVD‐organoids.^27^, ^28^

### 3.4 Reduced metabolic stress and improved cell survival in IVD‐organoids

**FIGURE 4:**
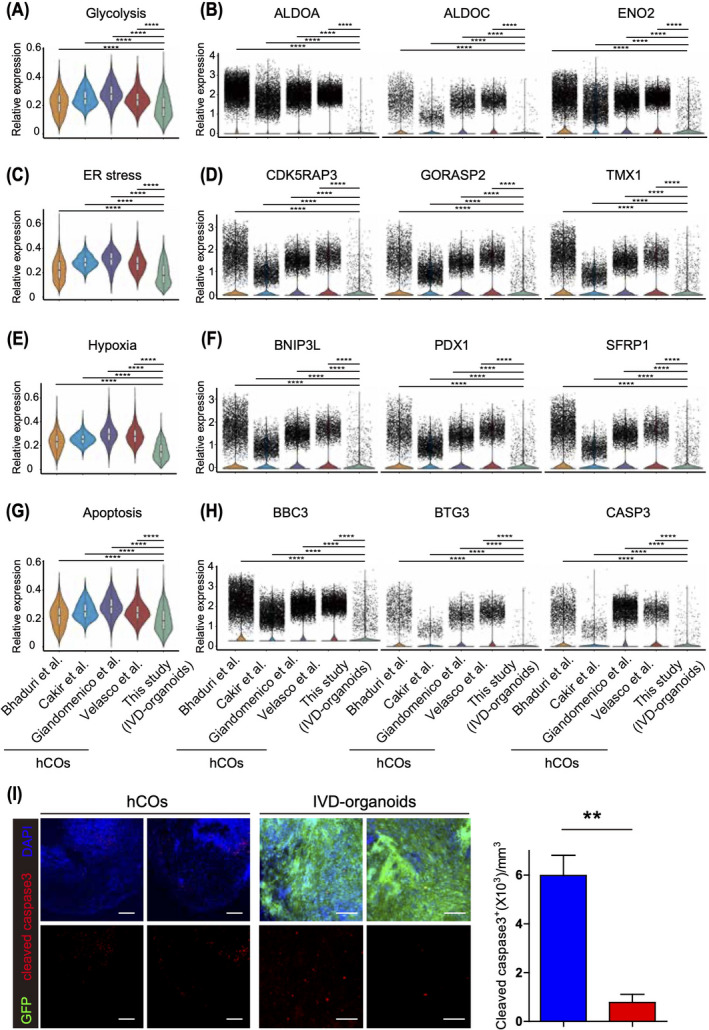
IVD‐organoids exhibit improved cell survival. (A) Glycolysis signature levels in IVD‐organoids and published hCOs. (B) Violin plots showing expression levels of glycolysis‐related genes. (C) ER stress signature levels in IVD‐organoids and published hCOs. (D) Violin plots showing expression levels of ER stress‐related genes. (E) Hypoxia signature levels in IVD‐organoids and published hCO. (F) Violin plots showing expression levels of hypoxia‐related genes. (G) Apoptosis signatures between IVD‐organoids and published hCOs. (H) Violin plots showing expression levels of apoptosis‐related genes. (I) Immunostaining of cleaved caspase 3 in IVD‐organoids and D60 hCOs. Scale bars, 200 μm. Quantification results are presented as mean ± s.e.m. (*n* = 3 for transplanted mice and cultured D60 hCOs). ∗∗*p* < 0.01, unpaired two‐tailed *t*‐test

It has recently been reported that metabolic pathways are often activated in organoids, leading to increased cellular stress in cultured hCOs.^18^ To compare metabolic stress between IVD‐organoids and hCOs, we performed integrated analysis of the common neuronal and neural progenitor cell types (i.e., neurons and RGCs) identified among IVD‐organoids and a few stage‐matched hCOs.^15^, ^18^, ^27^, ^29^ This analysis revealed that metabolic stress‐associated pathways, such as glycolysis, ER stress, and hypoxia, were significantly less activated in IVD‐organoids (Figure 4A, C, E). Indeed, we observed significant downregulation of key genes of these pathways in IVD‐organoids, including glycolysis‐related genes (ALDOA, ALDOC, and ENO2), ER stress‐related genes (CDK5RAP3, GORASP2, and TMX1), and hypoxia‐related genes (BNIP3L, PDX1, and SFRP1) (Figure 4B, D, F). In addition to the analysis of neuronal and neural progenitor cells, we next compared ChP cells in IVD‐organoids and those from in vitro ChP organoids^26^ and found that metabolic stress‐associated pathways were also less activated in ChP cells in IVD‐organoids (Figure S4A–C).

We next investigated the impact of in vivo microenvironment on the survival of IVD‐organoids. Since apoptosis pathways have been reported to be activated in long‐term cultured hCOs due to the lack of vasculature structures and brain microenvironment,^30^ we examined apoptosis signature genes neuronal and neural progenitor cells from either IVD‐organoids or cultured hCOs and found that apoptosis pathway was significantly less activated in IVD‐organoids (Figure 4G, H). Immunostaining of cleaved caspase 3 with IVD‐organoids and hCOs further confirmed these findings (Figure 4I). Similar observations were also made when comparing ChP cells from IVD‐organoids with those from in vitro cultured ChP organoids^26^ (Figure S4D). Together, our results indicate that the in vivo environment generally reduces metabolic stress and apoptosis during the formation of brain organoids.

## 4 DISCUSSION

The high fidelity of human brain organoids to primary tissue is an advantage for potential transplantation therapy. However, organoids are usually large in size and they require much larger surgical cavities for transplantation than single cells. The needles used in transplantation surgeries may also cause more severe surgical injury to healthy tissues along the entire route of organoid delivery. Thus, due to their large size, the transplantation of hCOs is limited to outer regions of the brain (e.g., the cerebral cortex) byzuot making an implantation cavity in the brain tissue. In this study, we generated human brain organoid in vivo by injecting small premature organoids with smaller needles, which was more suitable for the stereotaxic injection into deep brain structures with less surgical damage. Our results demonstrated that these premature organoids survived and continued to form organoid‐like structures. Further single‐cell transcriptome profiling also verified the cellular complexity in IVD‐organoids. By balancing the advantages and disadvantages of organoid and cell transplantation, our approach enables developing brain organoids in various brain regions including deep structures.

Vascularization of brain organoids is critical for their long‐term survival and functional maturation. It has been established that host‐mediated vascularization can be observed in organoid graft.^4^, ^5^ Similarly, host CD31‐positive blood vessels were found in our IVD‐organoids, suggesting that the integration of host vascular system improved IVD‐organoids survival. In our single‐cell transcriptome analysis, although we did not find any cell cluster containing CD31‐positive human endothelial cells, premature organoid‐derived cells with pericyte marker expression (PDGFRβ and COL3A1) were detected. Similar to our observation, pericytes were also identified in vascularized organoids.^15^ The presence of pericytes may facilitate graft survival and integration by promoting angiogenesis process as well as supporting the microvascular structures.^31^, ^32^ Although the most common developmental origin of pericytes is mesenchymal stem cells, studies also indicated their heterogenous origin which differs by tissues.^33^, ^34^, ^35^, ^36^ Opposed to a mesodermal origin, our results suggest an unexpected neuroectodermal ancestry of pericytes. It is intriguing to explore whether these neuroectoderm‐originated pericytes are functionally different from those derived from mesoderm. The ChP cells in IVD‐organoids were more mature compared with those in cultured hCOs. Since ChP epithelial cells express and secrete a variety of supportive factors for neuronal growth and survival, it is reasonable to speculate that the combination of host vascular system and these human ChP cells in IVD‐organoids can provide a more hospitable niche for organoid development and survival.^10^, ^11^, ^12^ Besides, compared with IVD‐organoids, more apoptotic cells accumulate in large mature organoids after a long period of in vitro culture due to lack of the vascular system.^37^, ^38^ Although these apoptotic cells are partially removed by microglia after transplantation, insufficient clearance (e.g., in elderly individuals) can lead to unwanted chronic immune response.^4^, ^39^, ^40^

Corpus striatum is one of the most vulnerable regions in neurodegenerative diseases and lacks endogenous neurogenesis, and thus a desired target site for cell therapy.^41^ However, it locates much deeper in the brain than cortex, which hampers the transplantation of large‐size hCOs. By generating IVD‐organoids in corpus striatum, new approaches for cell therapy may be developed in future. The majority of cells in in vitro cultured cerebral organoids using unguided methodologies express signature genes of cortical areas, including SATB2 and CTIP2.^2^, ^18^, ^42^ However, these markers only expressed in a small minority of cells in IVD‐organoids. Instead, striatal and ChP markers were widely expressed in IVD‐organoids. It is reported that the environment of transplantation sites including striatum could influence the differentiation and distribution of grafted cells, suggesting that the identity of IVD‐organoids may be regulated in a more complicated fashion, involving both the environmental cues (including but not limited to growth factors and neurotransmitters) from host striatum and intrinsic property of grafted cells.^43^, ^44^ Further investigation on IVD‐organoids in other host brain regions will lead to better understanding of the interplay between host brain and transplanted organoids.

## CONFLICT OF INTEREST

The authors declare no conflict of interest.

## AUTHOR CONTRIBUTIONS

G.P., L.S., and S.H. supervised this study. S.H. and Y.Y. performed organoid culture and stereotaxic surgery. J.L. performed immunostaining experiments. H.Z. and J.C. performed scRNA‐seq. F.H. and L.S. analyzed the scRNA‐seq data. S.H., F.H., L.S., and G.P. wrote the manuscript.

## Supporting information

Supplementary Material

## Data Availability

The GEO accession number for datasets reported in this study is GSE165975.

## References

[1] Budday S , Steinmann P , Kuhl E . Physical biology of human brain development. Front Cell Neurosci. 2015;9:257. 26217183 10.3389/fncel.2015.00257 PMC4495345

[2] Lancaster MA , Renner M , Martin CA , et al. Cerebral organoids model human brain development and microcephaly. Nature. 2013;501(7467):373‐379. 23995685 10.1038/nature12517 PMC3817409

[3] Qian X , Song H , Ming GL . Brain organoids: advances, applications and challenges. Development. 2019;146(8):dev166074. 30992274 10.1242/dev.166074 PMC6503989

[4] Mansour AA , Gonçalves JT , Bloyd CW , et al. An in vivo model of functional and vascularized human brain organoids. Nat Biotechnol. 2018;36(5):432‐441. 29658944 10.1038/nbt.4127 PMC6331203

[5] Daviaud N , Friedel RH , Zou H . Vascularization and engraftment of transplanted human cerebral organoids in mouse cortex. eNeuro. 2018;5(6):0219‐18. 10.1523/ENEURO.0219-18.2018 PMC6243198 30460331

[6] Kitahara T , Sakaguchi H , Morizane A , Kikuchi T , Miyamoto S , Takahashi J . Axonal extensions along corticospinal tracts from transplanted human cerebral organoids. Stem Cell Reports. 2020;15(2):467‐481. 32679062 10.1016/j.stemcr.2020.06.016 PMC7419717

[7] Shi Y , Sun L , Wang M , et al. Vascularized human cortical organoids (vOrganoids) model cortical development in vivo. PLoS Biol. 2020;18(5):e3000705. 32401820 10.1371/journal.pbio.3000705 PMC7250475

[8] Dong X , Xu SB , Chen X , et al. Human cerebral organoids establish subcortical projections in the mouse brain after transplantation. Mol Psychiatry. 2021;26(7):2964‐2976. 33051604 10.1038/s41380-020-00910-4 PMC8505255

[9] Chen HI , Wolf JA , Blue R , et al. Transplantation of human brain organoids: revisiting the science and ethics of brain chimeras. Cell Stem Cell. 2019;25(4):462‐472. 31585092 10.1016/j.stem.2019.09.002 PMC7180006

[10] Aliaghaei A , Khodagholi F , Ahmadiani A . Conditioned media of choroid plexus epithelial cells induces Nrf2‐activated phase II antioxidant response proteins and suppresses oxidative stress‐induced apoptosis in PC12 cells. J Mol Neurosci. 2014;53(4):617‐625. 24488602 10.1007/s12031-014-0228-4

[11] Lun MP , Monuki ES , Lehtinen MK . Development and functions of the choroid plexus‐cerebrospinal fluid system. Nat Rev Neurosci. 2015;16(8):445‐457. 26174708 10.1038/nrn3921 PMC4629451

[12] Watanabe Y , Matsumoto N , Dezawa M , Itokazu Y , Yoshihara T , Ide C . Conditioned medium of the primary culture of rat choroid plexus epithelial (modified ependymal) cells enhances neurite outgrowth and survival of hippocampal neurons. Neurosci Lett. 2005;379(3):158‐163. 15843055 10.1016/j.neulet.2004.12.068

[13] Kamouchi M , Ago T , Kitazono T . Brain pericytes: emerging concepts and functional roles in brain homeostasis. Cell Mol Neurobiol. 2011;31(2):175‐193. 21061157 10.1007/s10571-010-9605-x PMC11498428

[14] Xiang Y , Tanaka Y , Cakir B , et al. hESC‐derived thalamic organoids form reciprocal projections when fused with cortical organoids. Cell Stem Cell. 2019;24(3):487‐497.e487. 30799279 10.1016/j.stem.2018.12.015 PMC6853597

[15] Cakir B , Xiang Y , Tanaka Y , et al. Engineering of human brain organoids with a functional vascular‐like system. Nat Methods. 2019;16(11):1169‐1175. 31591580 10.1038/s41592-019-0586-5 PMC6918722

[16] Yu G , Wang L‐G , Han Y , He Q‐Y . clusterProfiler: an R package for comparing biological themes among gene clusters. OMICS. 2012;16(5):284‐287. 22455463 10.1089/omi.2011.0118 PMC3339379

[17] Evans AE , Kelly CM , Precious SV , Rosser AE . Molecular regulation of striatal development: a review. Anat Res Int. 2012;2012:106529. 22567304 10.1155/2012/106529 PMC3335634

[18] Bhaduri A , Andrews MG , Mancia Leon W , et al. Cell stress in cortical organoids impairs molecular subtype specification. Nature. 2020;578(7793):142‐148. 31996853 10.1038/s41586-020-1962-0 PMC7433012

[19] Guemez‐Gamboa A , Coufal NG , Gleeson JG . Primary cilia in the developing and mature brain. Neuron. 2014;82(3):511‐521. 24811376 10.1016/j.neuron.2014.04.024 PMC4104280

[20] Bell RD , Winkler EA , Sagare AP , et al. Pericytes control key neurovascular functions and neuronal phenotype in the adult brain and during brain aging. Neuron. 2010;68(3):409‐427. 21040844 10.1016/j.neuron.2010.09.043 PMC3056408

[21] Sakaguchi H , Kadoshima T , Soen M , et al. Generation of functional hippocampal neurons from self‐organizing human embryonic stem cell‐derived dorsomedial telencephalic tissue. Nat Commun. 2015;6:8896. 26573335 10.1038/ncomms9896 PMC4660208

[22] Trujillo CA , Gao R , Negraes PD , et al. Complex oscillatory waves emerging from cortical organoids model early human brain network development. Cell Stem Cell. 2019;25(4):558‐569 e557. 31474560 10.1016/j.stem.2019.08.002 PMC6778040

[23] Daviaud N , Chevalier C , Friedel RH , Zou H . Distinct vulnerability and resilience of human neuroprogenitor subtypes in cerebral organoid model of prenatal hypoxic injury. Front Cell Neurosci. 2019;13:336. 31417360 10.3389/fncel.2019.00336 PMC6682705

[24] Sivitilli AA , Gosio JT , Ghoshal B , et al. Robust production of uniform human cerebral organoids from pluripotent stem cells. Life Sci Alliance. 2020;3(5):e202000707. 32303588 10.26508/lsa.202000707 PMC7167289

[25] Narita K , Takeda S . Cilia in the choroid plexus: their roles in hydrocephalus and beyond. Front Cell Neurosci. 2015;9:39. 25729351 10.3389/fncel.2015.00039 PMC4325912

[26] Pellegrini L , Bonfio C , Chadwick J , Begum F , Skehel M , Lancaster MA . Human CNS barrier‐forming organoids with cerebrospinal fluid production. Science. 2020;369(6500):eaaz5626. 32527923 10.1126/science.aaz5626 PMC7116154

[27] Velasco S , Kedaigle AJ , Simmons SK , et al. Individual brain organoids reproducibly form cell diversity of the human cerebral cortex. Nature. 2019;570(7762):523‐527. 31168097 10.1038/s41586-019-1289-x PMC6906116

[28] Shah K , Bedi R , Rogozhnikov A , et al. Optimization and scaling of patient‐derived brain organoids uncovers deep phenotypes of disease. bioRxiv. 2020(2020):2026.

[29] Giandomenico SL , Mierau SB , Gibbons GM , et al. Cerebral organoids at the air‐liquid interface generate diverse nerve tracts with functional output. Nat Neurosci. 2019;22(4):669‐679. 30886407 10.1038/s41593-019-0350-2 PMC6436729

[30] Quadrato G , Nguyen T , Macosko EZ , et al. Cell diversity and network dynamics in photosensitive human brain organoids. Nature. 2017;545(7652):48‐53. 28445462 10.1038/nature22047 PMC5659341

[31] Attwell D , Mishra A , Hall CN , O'Farrell FM , Dalkara T . What is a pericyte? J Cereb Blood Flow Metab. 2016;36(2):451‐455. 26661200 10.1177/0271678X15610340 PMC4759679

[32] Brown LS , Foster CG , Courtney JM , King NE , Howells DW , Sutherland BA . Pericytes and neurovascular function in the healthy and diseased brain. Front Cell Neurosci. 2019;13:282. 31316352 10.3389/fncel.2019.00282 PMC6611154

[33] Yamazaki T , Mukouyama YS . Tissue specific origin, development, and pathological perspectives of pericytes. Front Cardiovasc Med. 2018;5:78. 29998128 10.3389/fcvm.2018.00078 PMC6030356

[34] Creazzo TL , Godt RE , Leatherbury L , Conway SJ , Kirby ML . Role of cardiac neural crest cells in cardiovascular development. Annu Rev Physiol. 1998;60:267‐286. 9558464 10.1146/annurev.physiol.60.1.267

[35] Dias Moura Prazeres PH , Sena IFG , Borges IDT , et al. Pericytes are heterogeneous in their origin within the same tissue. Dev Biol. 2017;427(1):6‐11. 28479340 10.1016/j.ydbio.2017.05.001 PMC6076854

[36] Yamazaki T , Nalbandian A , Uchida Y , et al. Tissue myeloid progenitors differentiate into pericytes through TGF‐beta signaling in developing skin vasculature. Cell Rep. 2017;18(12):2991‐3004. 28329690 10.1016/j.celrep.2017.02.069 PMC5393447

[37] Kelava I , Lancaster MA . Stem cell models of human brain development. Cell Stem Cell. 2016;18(6):736‐748. 27257762 10.1016/j.stem.2016.05.022

[38] Qian X , Su Y , Adam CD , et al. Sliced human cortical organoids for modeling distinct cortical layer formation. Cell Stem Cell. 2020;26(5):766‐781 e769. 32142682 10.1016/j.stem.2020.02.002 PMC7366517

[39] Koellhoffer EC , McCullough LD , Ritzel RM . Old maids: aging and its impact on microglia function. Int J Mol Sci. 2017;18(4):769. 10.3390/ijms18040769 PMC5412353 28379162

[40] Rock KL , Kono H . The inflammatory response to cell death. Annu Rev Pathol. 2008;3:99‐126. 18039143 10.1146/annurev.pathmechdis.3.121806.151456 PMC3094097

[41] Margulis J , Finkbeiner S . Proteostasis in striatal cells and selective neurodegeneration in Huntington's disease. Front Cell Neurosci. 2014;8:218. 25147502 10.3389/fncel.2014.00218 PMC4124811

[42] Kanton S , Boyle MJ , He Z , et al. Organoid single‐cell genomic atlas uncovers human‐specific features of brain development. Nature. 2019;574(7778):418‐422. 31619793 10.1038/s41586-019-1654-9

[43] Fjodorova M , Torres EM , Dunnett SB . Transplantation site influences the phenotypic differentiation of dopamine neurons in ventral mesencephalic grafts in Parkinsonian rats. Exp Neurol. 2017;291:8‐19. 28131726 10.1016/j.expneurol.2017.01.010 PMC5354310

[44] Quattrocolo G , Fishell G , Petros TJ . Heterotopic transplantations reveal environmental influences on interneuron diversity and maturation. Cell Rep. 2017;21(3):721‐731. 29045839 10.1016/j.celrep.2017.09.075 PMC5662128

