# Supplementary material for: In vivo development and single‐cell transcriptome profiling of human brain organoids
Source: Cell Prolif. 2022 Feb 10;55(3):e13201. doi: 10.1111/cpr.13201 (PMC8891563; doi:10.1111/cpr.13201)
Supplement: Supplementary file 1 — Supplementary Material [file CPR-55-e13201-s001.docx]

**supplementary INFORMATION**

**In vivo development and single-cell transcriptome profiling of human**

**brain organoids**

Shichao Huang^1,8*^, Fei Huang^2,8^, Huiying Zhang^2^, Yongfeng Yang^1^, Juan Lu^1^, Jiadong Chen^3^,

Li Shen^2,4,5*^, Gang Pei^1,6,7^

^1^ State Key Laboratory of Cell Biology, Shanghai Institute of Biochemistry and Cell Biology, Center for Excellence in Molecular Cell Science, Chinese Academy of Sciences, Shanghai 200031, China

^2^ The MOE Key Laboratory of Biosystems Homeostasis & Protection and Zhejiang Provincial Key Laboratory for Cancer Molecular Cell Biology, Life Sciences Institute, Zhejiang University, Hangzhou 310058, China

^3^ Center for Neuroscience and Department of Neurology of Second Affiliated Hospital, NHC and CAMS Key Laboratory of Medical Neurobiology, MOE Frontier Science Center for Brain Research and Brain-Machine Integration, School of Brain Science and Brain Medicine, Zhejiang University School of Medicine, Hangzhou, China

^4^ Department of Orthopedics Surgery, the Second Affiliated Hospital, School of Medicine, Zhejiang University, Hangzhou, China

^5^ Hangzhou Global Scientific and Technological Innovation Center, Zhejiang University (HIC-ZJU), Hangzhou, China;

^6^ Shanghai Key Laboratory of Signaling and Disease Research, Laboratory of Receptor-based Biomedicine, The Collaborative Innovation Center for Brain Science, School of Life Sciences and Technology, Tongji University, Shanghai, China

^7^ Institute for Stem Cell and Regeneration, Chinese Academy of Sciences, Beijing, China

^8^ Co-first authors

^*^ Correspondence: [huangshichao@sibcb.ac.cn](mailto:huangshichao@sibcb.ac.cn) (S.H.), [li_shen@zju.edu.cn](mailto:li_shen@zju.edu.cn) (L.S.)

**Supplementary Figures S1-S4**


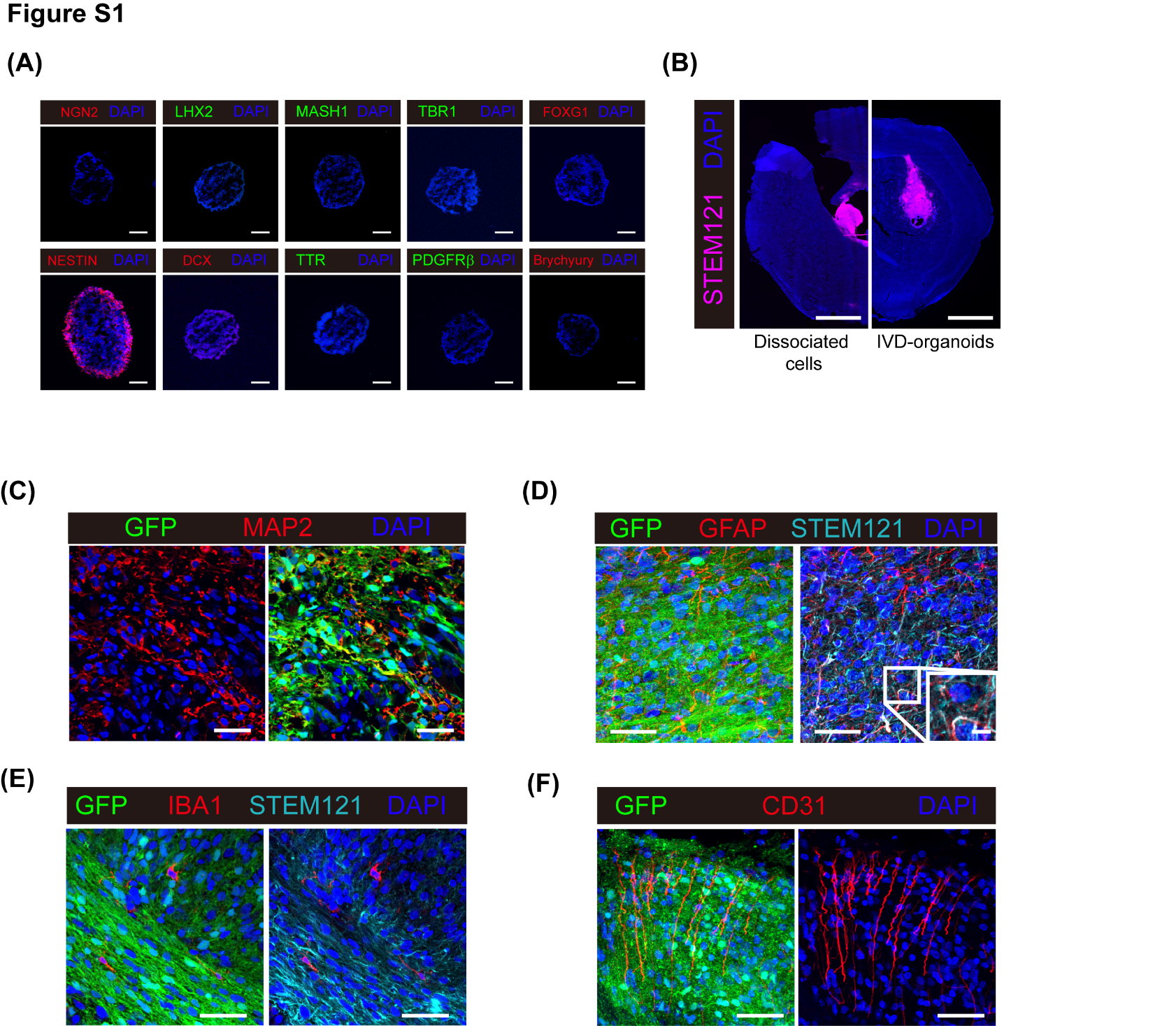


**Figure S1**

**Figure S1. Immunostaining of brain regions transplanted with dissociated cells.**

**(A)** Characterization of day-7 premature organoids with hippocampus marker (LHX2), MASH1, cortical marker (TBR1), FOXG1, neuronal markers (NGN2, NESTIN and DCX), ChP marker (TTR) and pericyte marker (PDGFRβ). Scale bar, 200 μm.

**(B)** Low magnification imaging of dissociated cells (left) and IVD-organoids (right). Scale bar, 1000 μm.

**(C)** Immunostaining of MAP2 in dissociated cells. Scale bar, 20 μm.

**(D)** Immunostaining of GFAP and STEM121 in dissociated cells. Scale bars, 50 μm and 10 μm (high-magnification imaging).

**(E)** Immunostaining of IBA1 in dissociated cells. Scale b­ar, 50 μm.

**(F)** Immunostaining of vascular marker CD31 in dissociated cells. Scale bar, 50 μm.

Related to Figure 1.


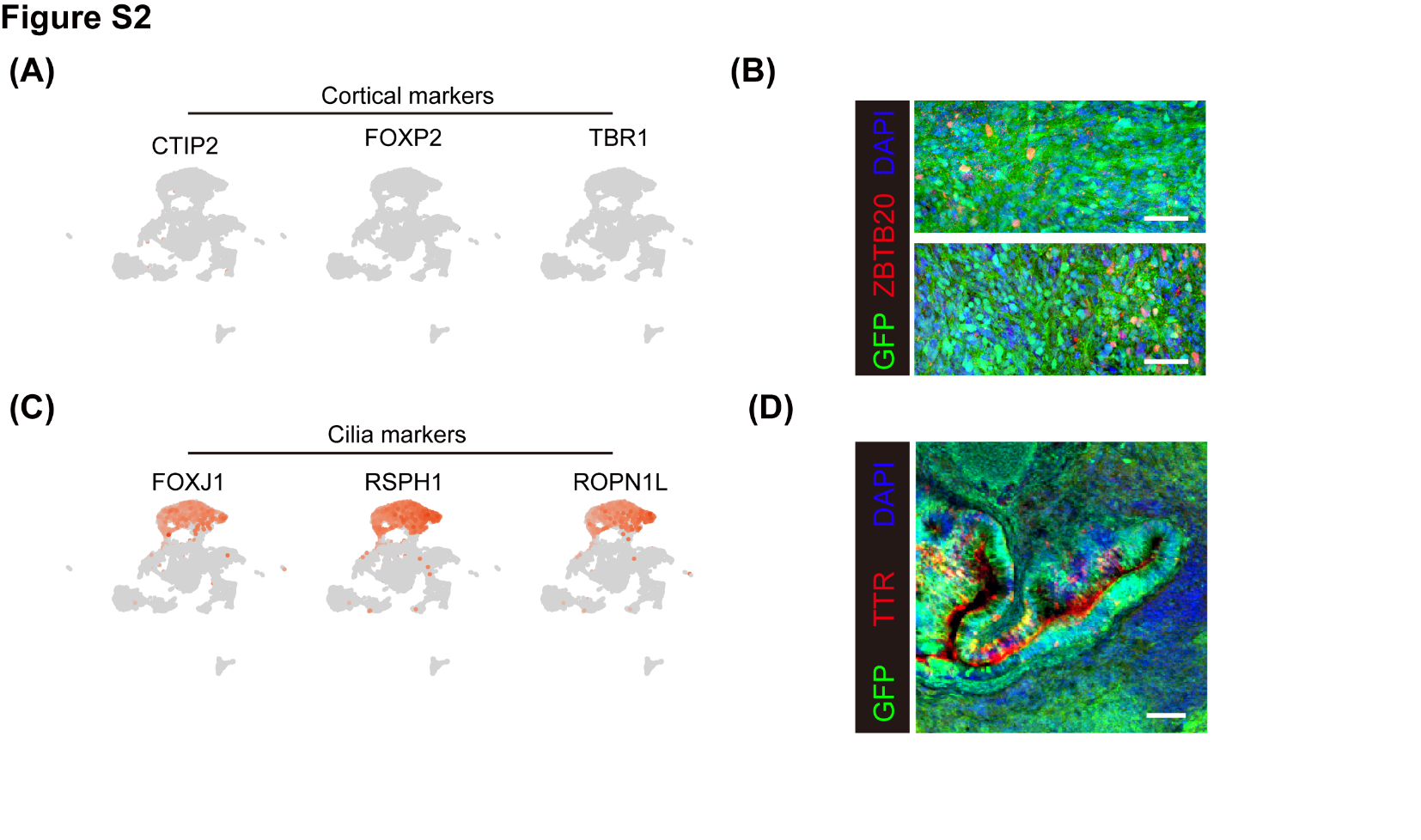
**Figure S2**

**Figure S2. Feature plots and immunostaining of various marker genes in IVD-organoids.**

(A) Feature plots of cortical markers in IVD-organoids.

(B) Immunostaining of ZBTB20 in IVD-organoids. Scale b­ar, 50 μm.

(C) Feature plots of cilia markers in IVD-organoids.

(D) Immunostaining of TTR in IVD-organoids. Scale b­ar, 100 μm.

Related to Figure 2.


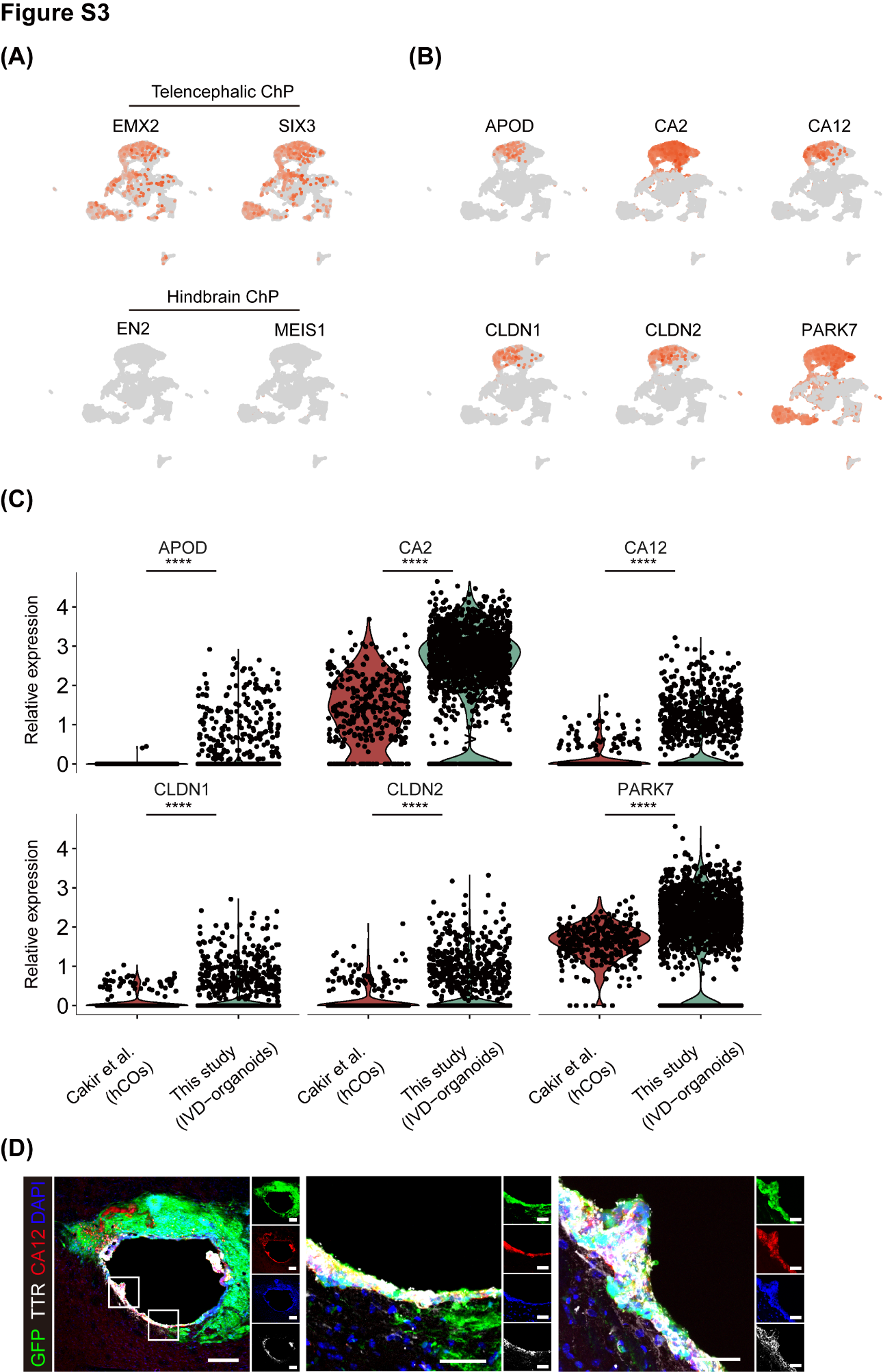
**Figure S3.**

**Figure S3. Integration analysis of IVD-organoids and hCOs (Cakir et al.).**

**(A)** Feature plots showing the expression patterns of telencephalic (top) and hindbrain (bottom) ChP markers in IVD-organoids.

**(B)** Feature plots showing the expression patterns of ChP-related genes (APOD, CA2, CA12，CLDN1, CLDN2 and PARK7) in IVD-organoids.

**(C)** Violin plots comparing ChP-related genes between the ChP clusters in IVD-organoids and hCOs.

(D) Immunostaining of CA12 and TTR in IVD-organoids. Scale b­ar in the left panel, 200 μm. Scale b­ars in the middle and right panel, 50 μm.

Related to Figure 3.

**Figure S4.**


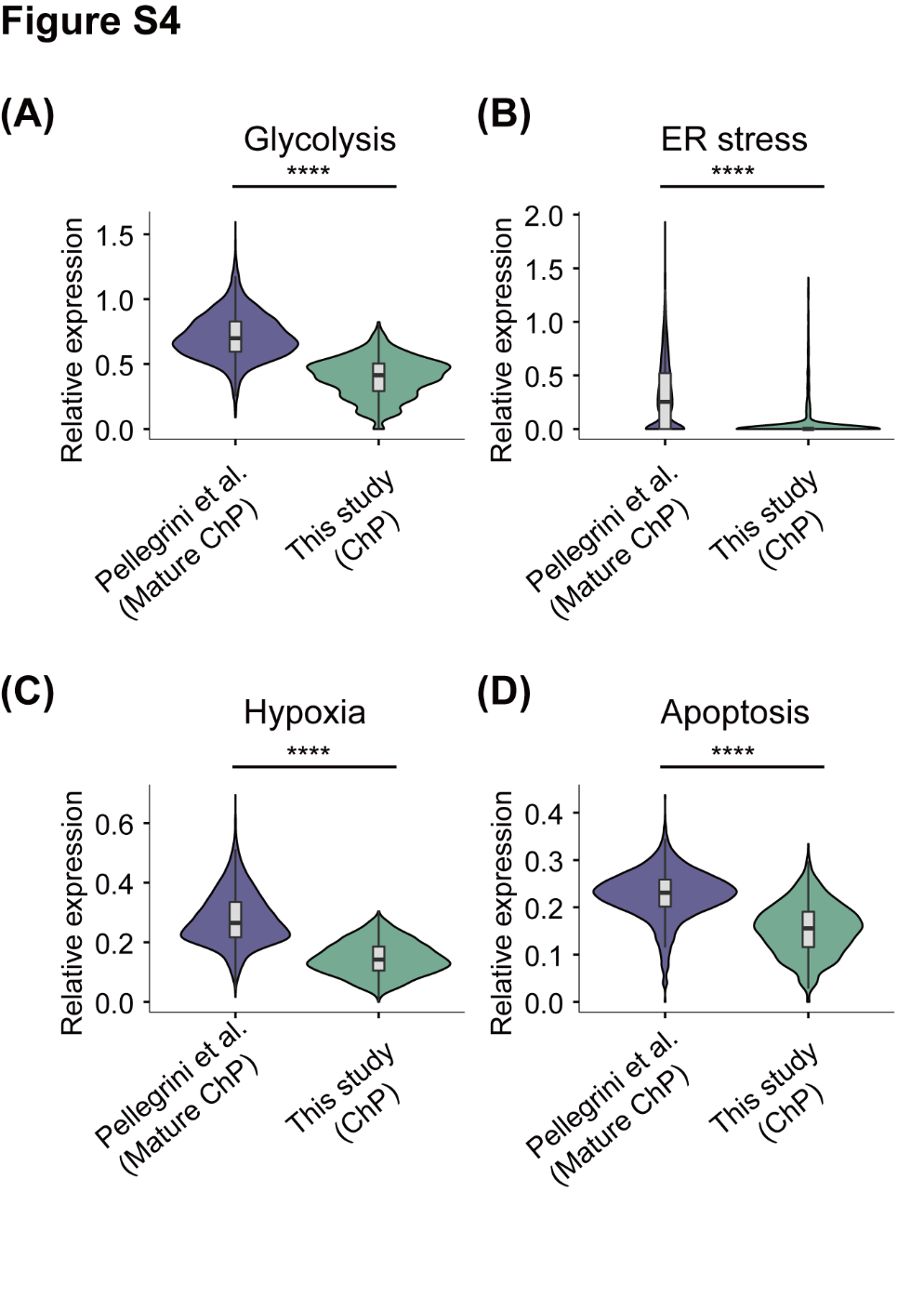


**Figure S4. Comparison of glycolysis (A), ER stress (B), hypoxia (C), and apoptosis (D) pathway levels between ChP cells in IVD-organoids and mature ChP cells in cultured ChP organoids.**

Related to Figure 4.
